# Digital transformation: artificial intelligence and employment anxiety of prospective sports managers

**DOI:** 10.3389/fpsyg.2025.1717674

**Published:** 2026-01-08

**Authors:** Zulbiye Kacay, Umran Sarikan, Hakan Sunay, Tuna Turgut, Laurentiu Gabriel Talaghir, Iconomescu Teodora Mihaela, Daniel Madalin Coja, Cristea Florentina

**Affiliations:** 1 Faculty of Sports Sciences, Çanakkale Onsekiz Mart University, Çanakkale, Türkiye; 2 Institute of Health Sciences, Ankara University, Ankara, Türkiye; 3 Faculty of Sports Sciences, Ankara University, Ankara, Türkiye; 4 Faculty of Sport Sciences, Bartın University, Bartın, Türkiye; 5 Faculty of Physical Education and Sport, Dunarea de Jos University of Galati, Galati, Romania

**Keywords:** sport, management, artificial intelligence, employment, anxiety

## Abstract

**Background:**

Digital transformation, a rapidly growing phenomenon in today’s business world, has brought profound changes across various sectors. In the field of sports management, its impacts are particularly significant, influencing prospective sports managers’ concerns about Artificial Intelligence (AI) and employment. To strengthen the theoretical grounding, recent research indicates that AI-driven automation is reshaping job roles, required competencies, and career expectations in sports-related professions. It is argued that sports management students are compelled to reshape both their professional skills and their job-seeking processes due to technological advancements in a digitalized world. In this context, the study aims to examine the concerns of prospective sports managers regarding AI and employment in the digital transformation era and provide practical recommendations.

**Methods:**

The research was conducted using a relational survey model. The study sample comprised of 210 individuals aged between 18 and 39 (Mean Age = 21.18), selected through convenience sampling. Data were collected using a personal information form prepared by the researchers, the “Artificial Intelligence Anxiety Scale,” and the “Employment Anxiety Scale for Sports Sciences Students.” Data analysis was performed using SPSS 24.0 software. Independent samples t-tests were used to assess differences, and Pearson correlation analysis was applied to determine relationships between variables. Effect sizes and assumption checks were also considered to strengthen interpretability (Cohen’s d, η^2^).

**Results:**

The findings revealed a significant difference in the mean scores for the “AI Configuration” sub-dimension of the AI Anxiety Scale based on gender. However, no significant differences were determined in the sub-dimensions of “Learning,” “Job Replacement,” and “Sociotechnical Blindness,” nor in the total scores of the Employment Anxiety Scale for Sports Sciences Students. Similarly, no significant differences were determined in the total scores and sub-dimensions of the AI Anxiety Scale or the total scores of the Employment Anxiety Scale based on age (ANOVA results). Income level, however, significantly affected the Employment Anxiety Scale scores, though no significant differences were observed for the total and sub-dimension scores of the AI Anxiety Scale.

**Conclusion:**

To alleviate employment anxiety among prospective sports managers, career counseling services and increased internship and job opportunities can be implemented. Economic support programs, such as scholarships and internship stipends, could help reduce insecurity among students from lower-income backgrounds. Furthermore, AI training programs may mitigate technological anxieties, enhancing students’ confidence in adapting to the digital transformation of their field.

## 1 Introduction

Digital transformation has emerged as one of the most influential dynamics reshaping the social, economic, and cultural structures of modern societies. At the core of this transformation lie Artificial Intelligence (AI) technologies, which have revolutionized numerous sectors by optimizing business processes, accelerating decision-making, and enhancing efficiency. Empirical studies demonstrate that AI adoption significantly alters job roles and competency demands across industries, including data-dependent fields such as sport management (Autor et al., 2022; Glebova et al., 2024). However, these advancements have also become a significant source of anxiety for individuals regarding job security, leading to psychological implications at the individual level. The potential of AI to take over routine tasks has deepened questions surrounding the value and functionality of human labor.

As a process affecting all domains, from the global economy to individual lives, digital transformation has brought fundamental changes to the field of sports management. AI technologies have become powerful tools in strategic planning, performance analytics, talent identification, injury prevention and fan engagement. Empirical studies demonstrate that AI adoption significantly alters job roles and competency demands across industries, including data-dependent fields such as sport management (Autor et al., 2022; Glebova et al., 2024). Alongside these opportunities, professional uncertainty and employment anxiety have emerged as critical concerns, particularly for prospective sports managers who must navigate changing skill expectations.

Prospective sports managers face the dual challenge of adapting to digitalization while AI related competencies and, simultaneously worry about how automation will affect employment opportunities. The transformations driven by AI in the labor market are reshaping the roles and responsibilities of sports managers, potentially reducing the demand for traditional administrative or routine skills. In this context, it is crucial for sports management candidates to align themselves with digital competencies and adopt a mindset of continuous learning. Therefore, understanding how digitalization influences students’ perceived competence and readiness is theoretically important and practically necessary (Christodimitropoulou et al., 2025). The employment anxieties triggered by AI-driven digital transformation can be conceptualized through two theoretical frameworks: Cognitive Behavioral Therapy (CBT) and Self-Determination Theory (SDT).

The employment anxieties stemming from the AI-focused changes brought about by digital transformation can be analyzed at an individual level through two key frameworks: Cognitive Behavioral Therapy (CBT) and Self-Determination Theory (SDT).

- Cognitive Behavioral Therapy (CBT): CBT focuses on understanding individuals’ negative thought patterns regarding AI and transforming these patterns into more functional approaches (Clark and Beck, 2010; Beck, 2011). It suggests that cognitive distortions, such as catastrophizing or overgeneralization about AI’s impact on employment, may heighten anxiety levels among prospective sports managers. CBT can help individuals recognize these distortions and develop more rational perspectives, reducing their anxiety related to technological disruptions.
- Self-Determination Theory (SDT): SDT emphasizes that attitudes and behaviors are shaped not by external pressures (e.g., societal expectations) but by intrinsic motivations, values, and individual decision-making (Çankaya, 2009). It identifies three innate psychological needs: autonomy, competence, and relatedness (Deci et al., 1996; Grolnick et al., 1997).
  - Autonomy involves feeling in control of one’s actions and decisions. The need for autonomy is related to the person feeling that she can decide for herself in her actions, rather than feeling that her actions are controlled or forced into them.
  - Competence refers to the need to feel capable of managing one’s environment.
  - Relatedness describes the need for meaningful and supportive social connections.

Guay et al. (2003) point out that it is necessary to meet these three basic needs in order to experience a sense of well-being and psychological development (Kocayörük, 2012).

Digital transformation and AI-related anxieties may threaten these fundamental needs, leading individuals to feel less competent or autonomous in their professional roles. For instance, the perception that traditional skills are becoming obsolete could erode feelings of competence, contributing to heightened employment anxiety.

Cognitive Behavioral Therapy (CBT) is a combination of behavioral therapy and cognitive therapy, which in its most basic form focus on the effects of thoughts, emotions (cognition) and behaviors that, when combined, are the source of psychological stress and disorders (Teater, 2015; Kök, 2024).

CBT and SDT provide a comprehensive framework to analyze the cognitive, emotional and motivational responses of prospective sports managers to digital transformation. When applied together, these approaches can facilitate healthier adaptation to change. CBT helps individuals confront and reframe irrational thoughts about AI’s impact, while SDT emphasizes fostering environments that support autonomy, competence and relatedness, enabling individuals to navigate technological shifts more confidently.

The aim of this study is to understand AI-induced employment anxieties among prospective sports managers, explore their psychological and structural determinants and propose solutions to mitigate them. By integrating CBT and SDT, the study offers a theoretically grounded perspective on how students cognitively and emotionally respond to digital transformation. Furthermore, this research contributes to ongoing discussions on how AI-driven shifts in the sports labor market (Glebova et al., 2024) and the expanding role of AI technologies in reshaping professional competencies and task structures in sport (Zhou et al., 2025) should inform curriculum design, student support systems and professional preparation in sport management education.

## 2 Methods

### 2.1 Participants

The research sample consisted of 210 volunteer students, aged between 18 and 39 (Mean age = 21.18), selected using the convenience sampling method. To improve transparency and replicability, inclusion criteria were: (a) being enrolled in a Sports Management program, (b) being an active student during the 2024–2025 academic year, and (c) providing informed consent. Students who submitted incomplete forms were excluded (*n* = 7). Participants were enrolled in the Sports Management Department of the Sports Sciences Faculties at Bartın University, Çanakkale Onsekiz Mart University, Ankara University, Van Yüzüncü Yıl University and Bolu Abant University during the 2024–2025 academic year.

A post-hoc power analysis conducted using G*Power 3.1 indicated that a minimum sample size of 176 was required to detect a medium effect size (*f* = 0.25) with 80% power at *α* = 0.05 for ANOVA; therefore, the sample size of 210 was considered adequate.

### 2.2 Research model

This study was conducted based on the quantitative research model, using correlational survey design. The correlational survey model is employed to identify relationships between two or more variables and to provide clues regarding cause-and-effect relationships (Karasar, 2020; Büyüköztürk et al., 2021). This design was selected because the primary aim was to examine associations between AI anxiety dimensions and employment anxiety without manipulating variables.

### 2.3 Data collection tools

The data collection process was conducted via an online form designed to include the scales and demographic questions. Prior to data collection, permission to use each scale was obtained from the original developers or adaptation authors. After obtaining the necessary permissions for the use of the scales, the study was carried out.

- Personal Information Form: Prepared by the researchers, this form includes demographic questions such as age, gender, class level, income status and the usage of Artificial Intelligence (AI) applications.
- Sports Sciences Students’ Job Finding Anxiety Scale: This unidimensional scale, developed by Aslan and Uğraş (2021), consists of eight items measured on a five-point Likert scale (1 = Never True, 5 = Always True). Higher total scores indicate higher levels of anxiety. The scale does not include reverse-coded items. Its reliability was determined with a Cronbach’s Alpha coefficient of 0.95 in the original study and 0.91 in this research.
- Artificial Intelligence Anxiety (AIA) Scale: Originally developed by Wang and Wang (2019) and adapted to Turkish by Akkaya et al. (2021), the scale comprises 16 items across four theoretically established sub-dimensions:

Learning (5 items): Reflects anxiety regarding acquiring knowledge or skills necessary to use AI tools.

Job Replacement (4 items): Captures concerns about AI potentially replacing human work roles.

Sociotechnical Blindness (4 items): Measures difficulties understanding or predicting AI-driven systems.

AI Configuration (3 items): Assesses anxiety related to operating and configuring AI processes.

These factor structures are derived from the original development and validation studies (Wang and Wang, 2019; Akkaya et al., 2021). Items are rated on a five-point Likert scale (1 = Strongly Disagree, 5 = Strongly Agree). The Cronbach’s Alpha coefficient for the scale in this study was 0.93 (Table 1).

**Table 1:** Reliability coefficients of scales.

Scales and dimensions	Items	Cronbach’s alpha
Learning	5	0.86
Job replacement	4	0.84
Sociotechnical blindness	4	0.90
AI Configuration	3	0.91
Artificial intelligence anxiety (AIA) scale	16	0.93
Sports sciences students’ job finding anxiety scale	8	0.91

In social sciences, Cronbach’s Alpha values above 0.70 are generally considered acceptable for internal consistency (Gürbüz and Şahin, 2015). All reliability coefficients exceeded this threshold, confirming satisfactory internal consistency for the scales and sub-dimensions.

### 2.4 Statistical analysis

The data were analyzed using the SPSS 24.0 software package. According to Haır et al. (2010), skewness and kurtosis values are recommended to assess the normal distribution of the data. George and Mallery (2003) suggest that data can be considered normally distributed if skewness and kurtosis values fall between +2 and −2. Based on this evaluation, the data in this study were determined to meet the criteria for normal distribution. In addition, Levene’s test was conducted to assess homogeneity of variances before applying t-tests and ANOVA.

Descriptive statistics, including mean and standard deviation (SD), were calculated. An independent samples t-test was employed to examine differences between two groups, while ANOVA was used to analyze differences among three or more groups. Pearson correlation analysis was applied to evaluate the relationships between mean scores of the scales. Correlation coefficients were interpreted as follows: 0.00: No relationship; 0.01–0.29: Low relationship; 0.30–0.70: Moderate relationship; 0.71–0.99: High relationship; 1.00: Perfect relationship (Köklü et al., 2007). Effect sizes were also calculated (Cohen’s *d* for *t*-tests; η^2^ for ANOVA) to provide a more comprehensive interpretation of group differences, as recommended in current statistical reporting standards. Given the number of subgroup comparisons, results were interpreted with attention to potential Type I error inflation, and adjusted *p*-values (Holm–Bonferroni) were considered in secondary analyses.

## 3 Results

The data revealed that among the participants, 96 were female (45.7%) and 114 were male (54.3%), with a mean age of 21.18. Regarding their academic level, 66 were first-year students, 9 were second-year, 67 were third-year and 68 were fourth-year students (Table 2).

**Table 2:** Demographic characteristics of the participants.

Demographic variable	Sub-group	Frequency	Percentage (%)
Gender	Female	96	45.7
	Male	114	54.3
Age	18–39	210 (x̄=21.18)	100.0
Grade	Grade 1	66	31.4
	Grade 2	9	4.3
	Grade 3	67	31.9
	Grade 4	68	32.4
How do you assess your monthly income?	Good	19	9.0
	Medium	147	70.0
	Low	44	21.0
Do you use artificial intelligence tools	Yes	141	67.1
	No	69	32.9

In terms of monthly income perception, 44 participants rated their income as poor, 147 rated it as moderate and 19 rated it as good. Additionally, 141 participants indicated that they use artificial intelligence tools, while 69 stated they do not (Table 6).

The independent samples *t*-test results showed a statistically significant difference only in the AI Configuration subdimension between female and male students [*t*(208) = 2.693, *p* = 0.008]. The effect size for this difference (Cohen’s *d* = 0.37) indicates a small-to-medium magnitude, suggesting that although female students report higher AI-Configuration anxiety, the practical impact of this difference is modest.

For the remaining subdimensions Learning, Job Replacement and Sociotechnical Blindness and for the total AI Anxiety and Employment Anxiety scores, no statistically significant gender differences were observed (all *p* > 0.05). The corresponding effect sizes (*d* = 0.05–0.20) were very small, and the 95% confidence intervals of group means showed substantial overlap. Therefore, these null findings should not be interpreted as definitive evidence of “no difference,” but rather as an indication that any potential gender effects are likely minor in this sample.

**Figure 1:**
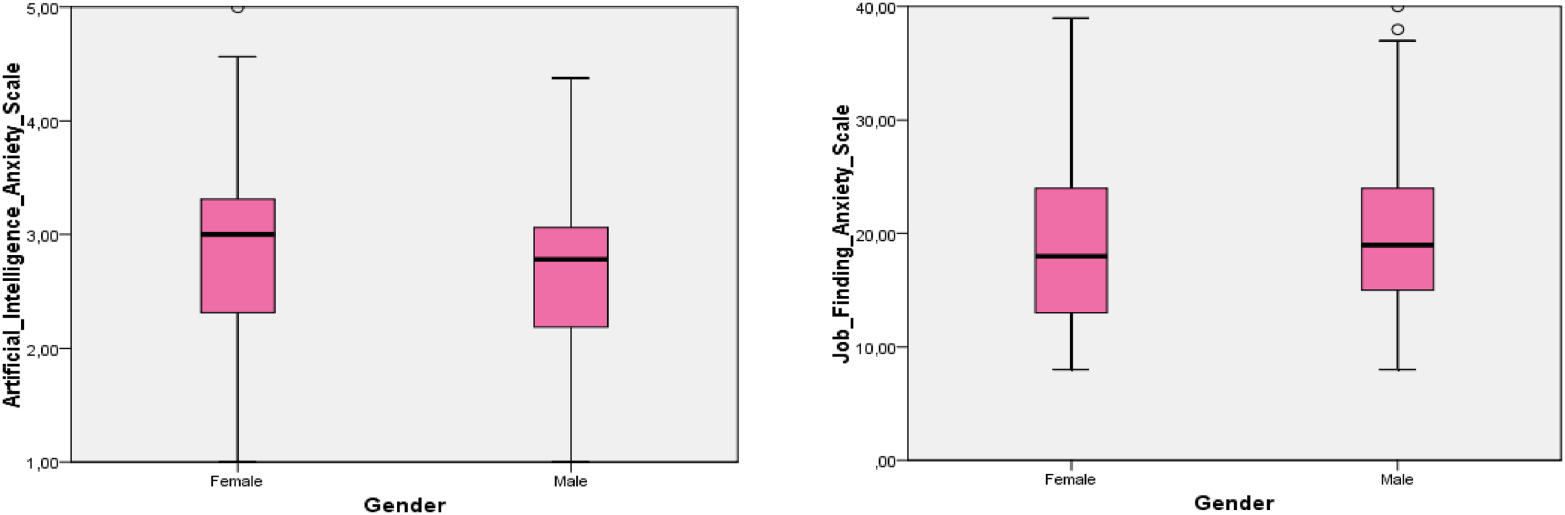
Boxplot of scale scores by gender.

Taken together, the findings suggest that gender plays a limited role in AI-related anxiety, with only a small effect emerging in the dimension related to operating/configuring AI systems. This pattern is consistent with prior research showing that gender differences in technology-related anxiety, when present, are typically small and context-dependent (Figure 1).

Upon examining the graphic, the boxplot illustrates detailed information on the scale scores (Job Finding Anxiety Scale and Artificial Intelligence Anxiety Scale) of sports sciences students, categorized by gender (Table 3).

**Table 3:** Independent samples *t*-test results regarding total scores of the artificial intelligence anxiety scale and its subdimensions, and the employment anxiety scale by gender.

Dimension	Variable	*n*	Mean	SS	SD	*t*	*P*
Learning	Female	96	11.906	3.482	208	1.130	0.260
	Male	114	11.377	3.289			
Job replacement	Female	96	11.927	3.806	208	1.492	0.137
	Male	114	11.175	3.487			
Sociotechnical blindness	Female	96	12.468	3.802	208	0.771	0.441
	Male	114	12.061	3.821			
AI Configuration	Female	96	9.166	3.080	208	2.693	**0.008**
	Male	114	8.017	3.080			
AIAS total	Female	96	45.468	11.971	208	1.771	0.078
	Male	114	42.631	11.216			
JFAS total	Female	96	18.635	6.940	208	−1.204	0.230
	Male	114	19.807	7.093			

*AI: Artificial Intelligence, AIAS: Artificial Intelligence Anxiety Scale, JFAS: Job Finding Anxiety Scale. *p < 0.05.

The ANOVA results indicated no statistically significant age-related differences in any of the AI Anxiety subdimensions (Learning, Job Replacement, Sociotechnical Blindness, AI Configuration) or in the total scores of the Artificial Intelligence Anxiety Scale and the Job Finding Anxiety Scale (all *p* > 0.05). The effect sizes for these comparisons were very small (η^2^ = 0.005–0.018), suggesting that age explained only a minimal proportion of variance in anxiety levels.

Moreover, the 95% confidence intervals around group means showed substantial overlap, indicating that even if small differences exist among the age groups, these differences are likely trivial in practical terms. Given that the sample consists primarily of young adults within a narrow developmental range (18–25), it is plausible that age does not meaningfully differentiate AI-related or employment-related anxiety in this population.

Therefore, these null findings should not be interpreted as evidence that age cannot influence such anxieties in other contexts, but rather that within this relatively homogeneous university-aged sample, age appears to play a limited role (Table 4).

**Table 4:** ANOVA results for total scores of the artificial intelligence anxiety scale and its subdimensions, and the job finding anxiety scale by age.

Dimension		Lines total	df	Lines average	*F*	*P*
Learning	Intergroup	156.484	12	13.040	1.150	0.322
	In group	2233.040	197	11.335		
	Total	2389.524	209			
Job replacement	Intergroup	126.398	12	10.533	0.782	0.669
	In group	2654.026	197	13.472		
	Total	2780.424	209			
Sociotechnical blindness	Intergroup	141.985	12	11.832	0.806	0.644
	In group	2891.138	197	14.676		
	Total	3033.124	209			
AI Configuration	Intergroup	168.338	12	14.028	1.475	0.136
	In group	1873.776	197	9.512		
	Total	2042.114	209			
AIAS total	Intergroup	1590.089	12	132.507	0.979	0.470
	In group	26661.839	197	135.339		
	Total	28251.929	209			
JFAS total	Intergroup	831.065	12	69.255	1.436	0.152
	In group	9502.464	197	48.236		
	Total	10333.529	209			

*AI: Artificial Intelligence, AIAS: Artificial Intelligence Anxiety Scale, JFAS: Job Finding Anxiety Scale. *p < 0.05.

The ANOVA results showed no statistically significant grade-level differences in the Job Replacement and Sociotechnical Blindness subdimensions or in overall employment anxiety (all *p* > 0.05). The effect sizes for these nonsignificant comparisons were very small (η^2^ = 0.01–0.02), and the 95% confidence intervals for group means largely overlapped, indicating that any potential grade-related differences in these domains are likely minimal.

However, significant differences emerged in the Learning (*F* = 2.999, *p* = 0.032), AI Configuration (*F* = 3.293, *p* = 0.022), and AIAS Total (*F* = 3.166, *p* = 0.025) scores. Although these effects were small to moderate in magnitude (η^2^ = 0.03–0.05), the pattern suggests that second-year students scored higher than students in more advanced grades.

This pattern may reflect differences in curricular exposure: earlier-year students typically encounter fewer digital tools and AI-related coursework, which may contribute to greater uncertainty and lower perceived competence in interacting with AI systems. As students progress to their third and fourth years, increased exposure to sport informatics, performance analysis technologies, and data-driven decision-making modules likely reduce anxiety by enhancing familiarity and capability.

These findings are consistent with Self-Determination Theory, which emphasizes the role of competence in reducing anxiety, and with recent research indicating that structured exposure to AI tools reduces confusion and apprehension among students (Zhou et al., 2025). Thus, grade-level differences in AI anxiety appear to be shaped more by differential exposure to digital learning experiences rather than by stable personal characteristics (Table 5).

**Table 5:** ANOVA results for total scores of the artificial intelligence anxiety scale and its subdimensions and the job finding anxiety scale by grade.

Dimension	Grade	*n*	Mean	SS	SD	*F*	*p*	Significance
Learning	Grade 1.^1^	66	11.727	3.510	3	2.999	0.032*	2-3-4
	Grade 2.^2^	9	14.777	5.118				
	Grade 3.^3^	67	11.373	2.999				
	Grade 4.^4^	68	11.338	3.197				
Job Replacement	Grade 1.^1^	66	11.818	3.802	3	1.911	0.129	–
	Grade 2.^2^	9	13.888	3.515				
	Grade 3.^3^	67	11.432	3.491				
	Grade 4.^4^	68	11.000	3.586				
Sociotechnical blindness	Grade 1.^1^	66	12.287	3.670	3	1.818	0.145	-
	Grade 2.^2^	9	14.888	3.370				
	Grade 3.^3^	67	12.328	3.677				
	Grade 4.^4^	68	11.779	4.043				
AI Configuration	Grade 1.^1^	66	8.666	2.973	3	3.293	0.022*	2-3
	Grade 2.^2^	9	11.333	2.692				
	Grade 3.^3^	67	7.970	2.948				
	Grade 4.^4^	68	8.617	3.327				
AIAS total	Grade 1.^1^	66	44.500	11.879	3	3.166	**0.025** ^*^	2-3
	Grade 2.^2^	9	54.888	12.859				
	Grade 3.^3^	67	43.104	10.597				
	Grade 4.^4^	68	42.735	11.653				
JFAS total	Grade 1.^1^	66	20.484	7.803	3	1.599	0.191	–
	Grade 2.^2^	9	21.555	8.545				
	Grade 3.^3^	67	18.179	5.882				
	Grade 4.^4^	68	18.867	6.988				

*p < 0.05.

The table indicates no significant differences in the total scores of the Artificial Intelligence Anxiety Scale and its subdimensions (Learning, Job Transition, Sociotechnical Blindness, and AI Configuration) based on income level. However, a statistically significant difference was determined in the total scores of the Job Finding Anxiety Scale among sports sciences students (*p* < 0.05) (Table 6).

**Table 6:** ANOVA results for total scores of the artificial intelligence anxiety scale and its subdimensions, and the job finding anxiety scale by income level.

Dimension	Income status	*n*	Mean	SS	SD	*F*	*p*	Significance
Learning	Good^1^	44	12.136	3.580	2	1.036	0.357	–
	Medium^2^	147	11.564	3.271				
	Low^3^	19	10.842	3.745				
Job replacement	Good^1^	44	11.818	3.300	2	2.002	0.138	–
	Medium^2^	147	11.632	3.605				
	Low^3^	19	9.947	4.415				
Sociotechnical blindness	Good^1^	44	12.863	3.800	2	1.367	0.257	–
	Medium^2^	147	12.204	3.701				
	Low^3^	19	11.157	4.549				
AI Configuration	Good^1^	44	9.045	3.018	2	0.787	0.456	–
	Medium^2^	147	8.442	3.085				
	Low^3^	19	8.157	3.685				
AIAS total	Good^1^	44	45.863	10.876	2	1.651	0.194	–
	Medium^2^	147	43.843	11.341				
	Low^3^	19	40.105	14.798				
JFAS total	Good^1^	44	15.181	5.682	2	14.129	0.000*	1-2-3
	Medium^2^	147	19.863	6.706				
	Low^3^	19	24.157	7.946				

*p < 0.05.

The table demonstrates that no significant differences were determined in the total scores of the Job Finding Anxiety Scale and the Artificial Intelligence Anxiety Scale (including its subdimensions: Learning, Job Transition, Sociotechnical Blindness, and AI Configuration) based on the usage of AI tools (*p* > 0.05) (Table 7).

**Table 7:** Independent samples *t*-test results for total scores of the artificial intelligence anxiety scale and its subdimensions, and the job finding anxiety scale by AI tools usage.

Dimension	Variable	*n*	Mean	SS	SD	*t*	*P*
Learning	Yes	141	11.354	2.966	208	−1.626	0.105
	No	69	12.159	4.071			
Job replacement	Yes	141	11.361	3.592	208	–0.893	0.373
	No	69	11.840	3.763			
Sociotechnical blindness	Yes	141	12.290	3.917	208	0.234	0.815
	No	69	12.159	3.604			
AI Configuration	Yes	141	8.539	3.074	208	–0.025	0.980
	No	69	8.550	3.251			
AIAS total	Yes	141	43.546	11.051	208	–0.681	0.497
	No	69	44.710	12.770			
JFAS total	Yes	141	19.241	7.154	208	–0.089	0.929
	No	69	19.333	6.824			

*p < 0.05.

As seen in the table, a moderate positive correlation was determined between the Learning subdimension and Job Transition (*r*[534] = *p* < 0.00), Sociotechnical Blindness (*r*[368] = *p* < 0.00), and AI Configuration (*r*[534] = *p* < 0.00) subdimensions of the Artificial Intelligence Anxiety Scale. Additionally, a strong positive correlation was observed between the Job Transition subdimension and Sociotechnical Blindness (*r*[787] = *p* < 0.00) and AI Configuration (*r*[671] = *p* < 0.00) subdimensions. Lastly, a strong positive correlation was determined between the Sociotechnical Blindness and AI Configuration subdimensions (*r*[716] = *p* < 0.00). However, no significant correlations were found between the subdimensions of the Artificial Intelligence Anxiety Scale and the total scores of the Job Finding Anxiety Scale among sports sciences students (Table 8).

**Table 8:** Correlation analysis results for subdimensions of the artificial intelligence anxiety scale and the job finding anxiety scale.

Variables		Learning	Job replacement	Sociotechnical blindness	AI configuration	JFAS total	AIAS total
Learning	R						
	P						
Job replacement	R	0.534^**^					
	P	0.000					
Sociotechnical blindness	R	0.368^**^	0.787^**^				
	P	0.000	0.000				
AI Configuration	R	0.439^**^	0.671^**^	0.716^**^			
	P	0.000	0.000	0.000			
JFAS total	R	−0.014	−0.104	−0.052	−0.045		
	P	0.836	0.132	0.454	0.512		
AIAS total	R	0.697**	0.907**	0.874**	0.842**	−0.066	
	P	0.000	0.000	0.000	0.000	0.340	1

*p < 0.05. **p < 0.01.

**Figure 2:**
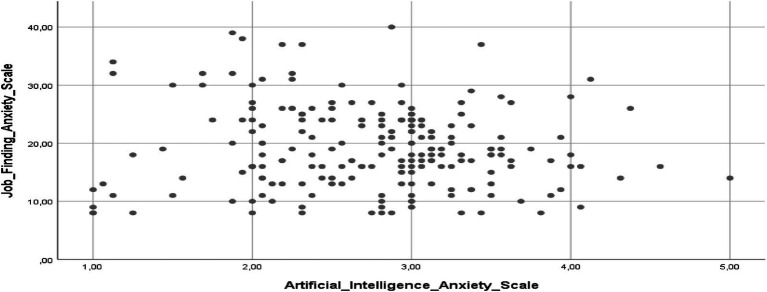
Scatter plot regarding relationships between scales.

Figure 2 provides detailed information on the relationship between the scales used in the study (Artificial Intelligence Anxiety Scale and Job Finding Anxiety Scale among sports sciences students).

## 4 Discussion

In this study, the job finding anxiety and Artificial Intelligence (AI) anxiety levels of prospective sports managers were analyzed in comparison with various variables and existing literature. The discussion integrates CBT and SDT perspectives to interpret the findings and situates the results within the broader context of digital transformation in the sports labor market.

The independent samples *t*-test results based on the gender variable revealed a significant difference in the mean scores of the AI Configuration subdimension, while no significant differences were observed in the Learning, Job Replacement, and Sociotechnical Blindness subdimensions or the total scores of the Job Finding Anxiety Scale among sports sciences students. Although the gender difference in AI Configuration anxiety was statistically significant, the effect size was small-to-moderate, indicating that the practical magnitude of this difference is limited. According to the literature, studies have reported that AI anxiety does not significantly differ based on gender (Sevimli Deniz, 2022). Although no statistical significance was determined in this study, it was observed that male participants generally exhibited higher levels of job finding anxiety compared to female participants. This may be attributed to societal expectations that place greater economic responsibility on men. However, the confidence intervals of group means overlapped substantially, suggesting that any gender-related differences in job-finding anxiety are likely to be small. This aligns with CBT’s emphasis on individual cognitive appraisals rather than demographic factors as determinants of anxiety. The literature also includes studies indicating significant differences in job finding anxiety based on gender (Yılmaz and Caz, 2022; Akdemir, 2021). On the other hand, consistent with this study, several other studies determined no significant difference in job finding anxiety concerning gender (Üngüren, 2007; Tümerdem, 2007; Demirci, 2020; Kuyulu et al., 2021; Turhan and Arslanboğa, 2022; Sarıkol and Ustaoğlu Hoşver, 2023). These findings suggest that equal opportunities and roles in the workplace for men and women may lead to similar levels of job anxiety. Contemporary evidence also suggests that AI-related competencies increasingly rely on skill-based rather than gender-based differences, reflecting broader changes in the digital sports industry (Zhou et al., 2025). In other words, in an environment where societal roles are equalized, job anxiety may become independent of gender.

The ANOVA results based on the age variable indicated no significant differences in the total scores of the AI Anxiety Scale and its subdimensions or the Job Finding Anxiety Scale among sports sciences students. This finding aligns with previous studies reporting no significant differences in job finding anxiety across age groups among university students (Bozyiğit and Gökbaraz, 2020; Şenel and Karakuş, 2022; Tutar and Öner, 2023). Because the sample is composed primarily of young adults within a narrow age range, age-based variance in AI anxiety or job-finding anxiety is expected to be minimal. The effect sizes in this study were also very small, indicating that any age-related differences are of limited practical importance.

Significant differences were identified in the Learning and AI Configuration subdimensions of the AI Anxiety Scale based on the class level variable, with second-, third-, and fourth-year students showing higher scores. This pattern suggests that anxiety may be shaped by differential exposure to AI-related content across years. First-year students may have limited familiarity with AI technologies, whereas upper-year students are more frequently exposed to digital tools, sport analytics modules, and applied technological coursework. No significant differences were found in the job finding anxiety scores of sports sciences students based on class level, consistent with literature findings indicating no significant effect of class level on job finding anxiety (Taşkent and Şahin, 2004). From an SDT perspective, students’ increasing exposure to AI-related coursework in later years may enhance their perceived competence, thereby reducing AI-related anxiety despite the rising complexity of academic requirements. These results also align with international research showing that structured exposure to AI increases perceived control and reduces uncertainty in sport management students (Zhou et al., 2025).

The ANOVA results based on income level revealed no significant differences in the total scores of the AI Anxiety Scale and its subdimensions. However, the literature suggests that university students with higher income levels tend to have lower AI anxiety scores (Aktaş Reyhan and Dağlı, 2023). In the present study, effect sizes for income differences in AI anxiety were very small, suggesting limited practical relevance. Conversely, significant differences were found in the Job Finding Anxiety Scale scores based on income level. Kaya (2022) reported that university students with lower income levels exhibited higher levels of job finding anxiety compared to those with higher incomes. Supporting these findings, another study by Tayfun and Korkmaz (2016) concluded that university students with low monthly income levels experienced higher unemployment anxiety. These results are consistent with broader literature indicating that socioeconomic vulnerability amplifies future-oriented anxiety. From a CBT viewpoint, perceived economic insecurity may reinforce negative automatic thoughts about employability, thereby increasing job-finding anxiety. This could be attributed to factors such as limited job opportunities, poor working conditions, job insecurity, and the perception of these factors as threats (Wichert, 2001).

Although AI anxiety and job-finding anxiety were conceptually expected to be related, no significant correlations emerged between these constructs. Rather than indicating the absence of any relationship, these null findings suggest that the two anxieties may operate through distinct psychological processes. AI anxiety may primarily reflect technology-specific cognitive and competence-related concerns, whereas job-finding anxiety is more closely linked to socioeconomic uncertainty. This interpretation is supported by evidence showing that AI-induced transformation of job roles in the sports industry affects required competencies but does not uniformly produce employment insecurity among students (Glebova et al., 2024).

## 5 Conclusion

Overall, the study’s findings highlight that AI-related concerns among sport management students are shaped more by exposure, competence perceptions, and educational experiences than by demographic characteristics alone. In contrast, job-finding anxiety appears to be more strongly influenced by socioeconomic vulnerability. These distinctions are important for designing targeted educational and counselling interventions.

In conclusion, this study examined the job finding anxiety and AI anxiety levels of prospective sports managers and evaluated the findings in relation to various variables. To reduce job finding anxiety among prospective sports managers, career counseling services can be offered, and internship and job opportunities can be increased. Economic support programs, such as scholarships and internship subsidies, can be provided for students with low-income levels to alleviate feelings of insecurity. Furthermore, AI-related training programs can be implemented to reduce technological anxieties, and career events that enhance interaction with the professional world can be organized. Psychological support and stress management services can also be provided to alleviate students’ anxieties. It is believed that these strategies could support the professional development of prospective sports managers while also reducing their job finding anxiety.

## Data Availability

The raw data supporting the conclusions of this article will be made available by the authors, without undue reservation.
